# A Systematic Review of Community-Level Protective Factors in Children Exposed to Maltreatment

**DOI:** 10.1177/15248380221117234

**Published:** 2022-09-01

**Authors:** Arianne Jean-Thorn, Amélie Tremblay-Perreault, Valéry Dubé, Martine Hébert

**Affiliations:** 1 Université du Québec à Montréal, Montréal, QC, Canada

**Keywords:** resilience, systematic review, maltreatment, protective factors, community factors

## Abstract

Childhood maltreatment and neglect are associated with a host of negative outcomes. Yet, some children show resilience despite their exposure to these traumatic events. Several protective factors have been documented in the literature, but few studies focus on protective factors in the child’s community that can promote resilience. The purpose of this review was to provide a comprehensive portrait of the impact of community protective factors on the resilience of abused and neglected children. The databases PsycNet and PubMed were used to screen the literature relying on the following inclusion criteria: (1) published in English or in French; (2) report empirical and quantitative data; (3) include a minimum sample size of 30 participants; (4) rely on a sample of maltreated children or adolescents under the age of 24; (5) examine the associations between community protective factors and indicators of psychological adaptation; and (6) include outcome measures that assessed either positive adaptation or the absence of symptomatology in participants. Of the 9,553 articles identified, 44 studies met the eligibility criteria for inclusion in this review. Although many protective factors show significant results, several methodological limitations remain to be examined to affirm that these community variables have a significant impact on the level of resilience of maltreated children. Since child maltreatment is a systemic issue, it remains important to fully understand how community protective factors operate on the resilience of these children as it can greatly inform practitioners and community institutions on how to intervene with populations at risk of maltreatment.

Child maltreatment is a global public health issue that requires the collaboration of several institutions to not only implement efficient prevention strategies, but also to document the associated consequences that emerge throughout the life span, such as a variety of deleterious outcomes including behavioral and emotional problems, post-traumatic stress disorder, depression, and anxiety (Hailes et al., 2019). Yet, studies conducted to date have shown that outcomes following child maltreatment are quite diverse and that not all children exhibit clinical levels of symptoms. Some children appear to follow a trajectory of resilience and show positive adaptation despite adversity (Yoon et al., 2020).

Resilience, defined here as the ability to adapt positively in the face of adversity, has been widely studied in the literature. In the last decades, the analysis of factors related to positive adaptation in the face of adversity has been further explored in the light of a systemic approach. Indeed, studies report that profiles of resilience in children who have been exposed to maltreatment are not only influenced by individual characteristics, but also by the attributes of the environment that surrounds them. A systemic view of resilience takes into consideration risk and protective factors at the individual, relational, and community levels that influence children’s level of resilience (Ungar, 2011). Theron and Ungar (2018) also argue that while focusing mainly on individual factors may narrow down prevention and intervention efforts, increasing our knowledge of external protective factors, such as schools, organized activities, as well as health and social services, could rather extend their scope. However, the bulk of studies has examined the impact of individual or interpersonal variables on resilience of maltreated children. Although the results emphasize the individual and internal capacities of resilient children, such a circumscribed approach insidiously places the burden of healing on the shoulders of vulnerable children (Theron & Ungar, 2018). Yet, the child’s broader environment and access to resources within that environment can indirectly influence individual abilities (Ungar et al., 2013). Some authors show that the collective efficacy of these community institutions can enhance or hinder children’s motivation or temperament (Beckett et al., 2006). Enhancing community resources (community services, institutions, and neighborhood) can potentially foster resiliency for many victimized youths, thus having a broader reach by being accessible to children in the community who may not have access to specialized services. Considering the impact of community factors over other types of factors, it is crucial to acknowledge the influence of the social ecology of maltreated children on their level of resilience.

While a systemic perspective would be beneficial to institutions working for the healthy development of children, there are only a few studies that have explored the impact of community factors on the resilience of maltreated children. A systematic review combining these community factors would be relevant to provide a comprehensive picture of the important elements of the social ecology of maltreated children.

Of the few available reviews that have considered community factors associated with resilience, the majority did not report any information on the measures used to assess protective factors and resilience, and/or only reported positive results without acknowledging nonsignificant results. Hence, these reviews did not provide a comprehensive overview of the literature. There is a need to focus exclusively on community factors to fully comprehend the state of the literature without allowing individual factors to overshadow the potential of external resources.

As individual and relational factors have been more widely studied, and as few studies examine community variables specifically, the current review aimed to provide a portrait of the impact of community protective factors on the resilience of maltreated and neglected children. This systematic review differs from previous reviews, notably by its neutrality in reporting both positive and negative results, so that readers can have an accurate picture of the state of knowledge. The objectives of this review were to (1) quantify the resilience rates of maltreated children and adolescents in the included studies; (2) identify community protective factors of resilience in maltreated youth and synthesize the results; (3) evaluate the quality of the studies following a standardized coding procedure and describe their methodological limitations. This systemic review aimed to update the state of knowledge on community protective factors that foster resilience of maltreated children and to highlight gaps in the literature. This work will direct efforts to operationalize the concept of resilience, to standardize and validate relevant assessment measures, and to promote rigorous methodology.

## Method

This systematic review was conducted using the Preferred Reporting Items for Systematic Reviews and Meta-Analyses (PRISMA) guidelines (Page et al., 2021).

### Inclusion and Exclusion Criteria

In order to be included in the sample, the studies had to (1) have been published in English or in French; (2) report empirical and quantitative data; (3) include a minimum sample size of 30 participants; (4) rely on a sample of maltreated children or adolescents under the age of 24; (5) examine the associations between community protective factors and indicators of psychological adaptation; and (6) include outcome measures that assessed either positive adaptation or the absence of symptomatology in participants. Studies were excluded if they only measured the effects of individual capacities or relational protective factors on the participant’s adaptation. To be considered community-level protective factors, variables had to relate to community institutions (e.g., schools, health and social services, community activities, and neighborhood). Therefore, variables that were of relational nature were included only if they pertained to an actor in one of these institutions. For instance, peer relationships were excluded because they were considered a relational factor in most of the studies. Although type of placement was often considered a familial factor in the studies examined, we chose to conceptualize it as a community factor since it is a function of the foster care system. Whilst informal support from an extrafamilial adult could also be considered an interpersonal factor, it was nonetheless included in this review in order to remain consistent with Meng et al.’s (2018) systematic review. We included participants up to 24 years old because (1) some study samples included an age range exceeding 18 years old, and (2) adolescence is now considered to last until the age of 24 (Sawyer et al., 2018).

There was no cut-off year of publication. In this review, we define child maltreatment as sexual abuse, physical, emotional, psychological maltreatment, or neglect (Jud et al., 2016). All forms of maltreatment were included, and studies were included whether multiple forms of maltreatment were examined altogether, or a single form was considered. The authors drew on a recent key systematic review (Meng et al., 2018) and on the fact that most articles on community factors were conducted in the United States, to define maltreatment. Also, considering that the definition of the Child Maltreatment Surveillance: Uniform Definitions for Public Health and Recommended Data Elements does not include domestic violence, studies conducted with participants only exposed to intimate partner violence were excluded (Leeb et al., 2008). All types of reports of maltreatment were included (caseworker reports, parent report, etc.). Articles were excluded from the review if they were commentaries, reviews, or qualitative studies. Studies were also excluded if it was impossible to distinguish whether participants had experienced maltreatment or adverse childhood experiences, which is a broader term encompassing adverse events that do not strictly fall under the definition of maltreatment. Studies examining only physical health outcomes were excluded.

### Data Sources and Search Strategy

**Figure 1.**
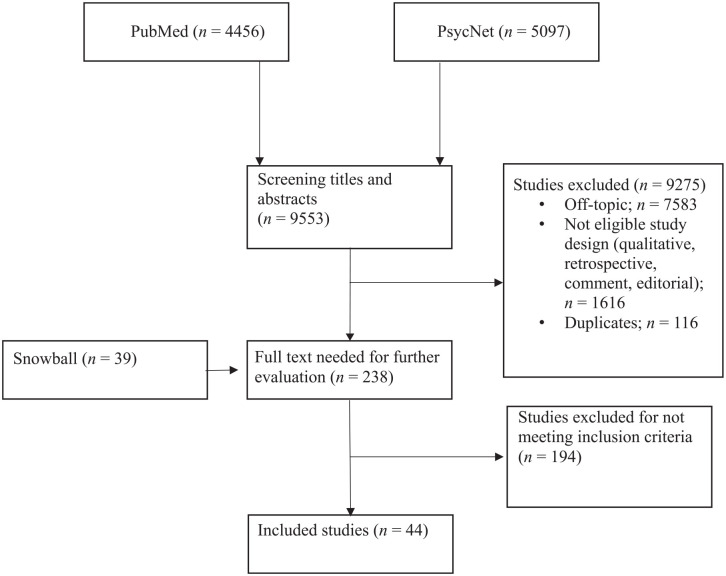
Summary of study selection process.

Between December 2020 and January 2021, PsycNet, which includes PsycArticle and PsycInfo, and PubMed were examined to identify key studies that met the inclusion criteria. The snowball technique was used when reading reviews or empirical articles to identify additional studies. Search strategy is detailed in Appendix A. The eligibility was done independently in an unblinded and standardized process by the first two authors. Uncertainties and disagreements between the two authors were resolved by consensus. An overview of the selection process is presented in Figure 1. All studies were coded with an inter-rater procedure. The inter-rater agreement was 94%.

### Quality Assessment

A data extraction sheet was created to collect information of each eligible study. Quality was assessed based on the *Standard Quality Assessment Criteria for Evaluating Primary Research Papers* (Kmet et al., 2004). Quality assessment is described in Appendix A. The assessment of the articles was to ensure their quality and clarity regarding the objectives, methodology, and results in terms of replicability and generalizability of the study. The quality of the methodology was assessed by the consistency between the definition of resilience and the choice of measures as well as between the sample size and the number of variables. The quality of the analyses and results was determined, amongst other components, by the fact that variance estimates were reported, and confounders were controlled for. The first two authors of the present review independently coded these criteria, and discrepancies were resolved through consensus (Kmet et al., 2004). According to the results on each of the criteria, every study included in the sample was coded from *Low* to *High* levels of potential bias. The study was rated *Low* if the methodology was rigorous and few risks of bias were identified. Studies were rated *Moderate* if some biases were impossible to ignore, but these would not drastically influence the results. The study was rated *High* if several risks of bias were identified in the methodology. The study was rated *Unclear* if essential information was missing to adequately judge the quality of the study. The characteristics of each study as well as the risk of bias are presented in Table 1.

**Table 1.** Summary of Studies Included in This Review and Protective Factors (*n* = 44).

Study	Sample		Maltreatment type	Community variables and measures used	Risk of bias
Cross-sectional studies					
Asgeirsdottir et al. (2010)	Iceland	*n* *=* 9,113 (age: 16–19), 51% girls	Sexual abuse	Sport participation,^1^ attitude toward school^1^	High
Aydin et al. (2016)	Turkey	*n* *=* 182 (age: 6–18), 87.4% girls	Sexual abuse	Teacher social support^1^	High
Chandy et al. (1996)	USA	*n* = 2,022 (*M*_age_ = 15.28), 100% girls	Sexual abuse	Clinic/nurse in school^1^	High
Dang (2014)	USA	*n* = 150 (age: 14–21), 57% girls	Physical abuse; sexual abuse (not distinguished; among homeless youth)	School connectedness^1^	Low
Edmond et al. (2006)	USA	*n* = 99 (age: 15–18), 100% girls	Sexual abuse	School stability,^1^ attendance to religious services^1^	Moderate
Eisenberg et al. (2007)	USA	*n* = 83,731 (6th–12th graders), 51.1% girls	Sexual abuse	Teacher and other adult support,^1^ school safety^1^	High
Emery et al. (2015)	Vietnam	*n* = 509 (*M*_age_ = 12.04), 85.77% girls	Physical abuse	Perceived collective efficacy,^2^ informal social control of child maltreatment^2^	Low
Ezzell et al. (2000)	USA	*n* = 37 (age: 6–14), 44% girls	Physical abuse	Teacher social support^1^	High
Feiring et al. (1998)		*n* = 154 (age: 8–15), 75% girls	Sexual abuse	Social support by nonrelative adult^1^	Moderate
Flores et al. (2005)^a^	USA	*n* = 133 (age: 6–8), 30.8% girls	All (not distinguished)	Extrafamilial caring adult^3^	Low
Go et al. (2017)	Singapore	*n* = 130 (age: 13–19), 53% girls	Neglect; physical abuse; emotional abuse (not distinguished)	Educational support^4^	High
Hildebrand et al. (2019)	Brazil	*n* = 166 (age: 9–16), 53.6% girls	All (not distinguished)	Teacher social support^1^	High
Kassis et al. (2018)	Austria, Germany, Slovenia, Spain	*n* = 5,149 (8th graders), 47% girls	Physical abuse	Relations with teachers^1^	Moderate
Kim (2008)^a^	USA	*n* = 384 (age: 6–12), 48% girls	All (not distinguished)	Attendance to religious services^1^	Low
Martinez-Torteya et al. (2017)^d^	USA	*n* = 597 (age: 12), 57% girls	Physical abuse; neglect; sexual abuse; exposure to violence (not distinguished).	Neighborhood quality^2^	Moderate
Maschi et al. (2010)	USA	*n* = 300 (age: 5–11), 37% girls	All (not distinguished)	Neighborhood safety^1^	Moderate
Pérez-González et al. (2017)	Spain	*n* = 1,105 (age: 12–17), 47% girls	Sexual victimization	School supportive environment,^1^ community connectedness^1^	Low
Perkins and Jones (2004)^b^	USA	*n* = 16,313 (age: 12–17), 53.3% girls	Physical abuse	Other adult support,^1^ school climate,^1^ extracurricular activities^1^	High
Perkins et al. (2002)^b^	USA	*n* = 18,592 (age: 12–18), 100% girls	Physical abuse	Other adult support,^1^ school climate^1^	High
Pharris et al. (1997)	USA	*n* = 13,923 (7th–12th graders), 50.7% girls	Sexual abuse	Caring from an adult,^1^ involvment in traditional activities^1^	High
Sagy and Dotan (2001)	Israel	*n* = 226 (8th graders), 58.4% girls	All (not distinguish)	School membership^1^	High
Sianko et al. (2016)	USA	*n* = 589 (age: 10–17), 51.4% girls	Family violence	Neighborhood safety,^1^ community services^1^	Moderate
Williams and Nelson-Gardell (2012)^c^	USA	*n* = 237 (age: 11–16), 80% girls	Sexual abuse	Neighborhood safety^2^	Low
Yonas et al. (2010)^d^	USA	*n* = 786 (age :12), 50% girls	Physical abuse; sexual abuse; neglect (distinguished)	Collective efficacy^2^	Low
Yoon et al. (2017)^c^	USA	*n* = 499 (age: 4–5), 48% girls	All (distinguished)	Neighborhood safety^2^	Low
Longitudinal studies					
Chapple and Vaske (2010)	USA	*n* = 1,080 (age: 2–13), 51% girls	Educational, physical and emotional neglect	Neighborhood organization/climate,^2^ school organization/climate^2^	Moderate
Choe (2021)	South Korea	*n* = 1,316 (7th–9th graders), 50.8% girls	Neglect	Teacher relationships^1^	High
Cicchetti and Rogosh (1997)^a^	USA	*n* = 213 (age: 6–11), 37.6% girls	All (not distinguished)	Camp counselor relationship^3^	Moderate
Daigneault et al. (2007)	Canada	*n* = 86 (age: 11–17), 100% girls	Sexual abuse	Out of home placement^1^	High
Holmes et al. (2018)^c^	USA	*n* = 1,776 (age: 0–10), 47% girls	All (distinguished)	Neighborhood quality^2^	Low
Jaffee et al. (2007)	England	*n* = 2,181 (age: 5–7), 51% girls	Physical abuse	Neighborhood characteristics,^2^ informal social control,^2^ social cohesion^2^	High
Khambati et al. (2018)	UK	*n* = 1,493 (age: 0–18), 77% girls	Emotional abuse Physical abuse (distinguished)	Extracurricular activities^2^	High
Kwak et al. (2018)^c^	USA	*n* = 790 (age: 12–16), 59% girls	All (distinguished)	Activity participation^1^	Low
Lemkin et al. (2018)^d^	USA	*n* = 318 (age: 4–18), 57.5% girls	All (distinguished)	Extracurricular activities,^1^ supportive adult at school^1^	Moderate
Leon et al. (2008)	USA	*n* = 142 (age: 10–17), 27% girls	Sexual abuse; physical abuse (distinguished; with problematic sexual behaviors)	Clubs,^2^ support from caseworker agency^2^	High
Lutman and Farmer (2013)	England	*n* = 138 (age: 0–14), 41% girls	Neglect	Placement stability,^4^ specialist support^4^	High
Oshri et al. (2017)^c^	USA	*n* = 1,179 (age: 11–15), 57.93% girls	All (not distinguished)	Community environment,^2^ service provision^4^	Low
Pittenger et al. (2018)^c^	USA	*n* = 1,664 (age: 11–17), 61.9% girls	Neglect, physical abuse, sexual abuse, or other (distinguished)	Attendance to religious services^1^	Low
Proctor et al. (2010)^d^	USA	*n* = 279 (age: 6–14), 53.4% girls	All (distinguished)	Caretaker stability^2^	Moderate
Sasser et al. (2019)^d^	USA	*n* = 1,354 (age: 4–18), 52.40% girls	All (not distinguished; who experienced bereavement)	Neighborhood collective efficacy^1^	Low
Sattler and Font (2018)^c^	USA	*n* = 1,193 (age: 0–14), 51% girls	All (not distinguished)	Neighborhood quality^2^	UC
Taussig (2002)	USA	*n* *=* 110 (age: 7–12), 60% girls	All (not distinguished)	Teacher social support^1^	UC
Yoon et al. (2018)^c^	USA	*n* = 350 (age: 11–13), 53.2% girls	All (distinguished)	Neighborhood safety^2^	Low
Yoon et al. (2020)^c^	USA	*n* = 711 (age: 11–17), 56.8% girls	All (distinguished)	Neighborhood safety,^2^ type of placement,^4^ receipt of behavioral services^4^	Moderate

*Note.* UC = unclear.

a Summer camp study.

b Search Institute’s Profiles of Student Life: Attitudes and Behaviors survey.

c National Survey of Child and Adolescent Well-Being I or II (NSCAW I or II).

d Longitudinal Studies on Child Abuse and Neglect (LONGSCAN).

1 Self-report.

2 Parent/caregiver report.

3 Teacher/counsellor report.

4 Clinical/educational file or caseworker report.

**Table:** *Critical findings*.

**Critical findings**
• In the 44 studies eligible for this review, the prevalence of resilience ranged from 1.5% to 66.7%, thus revealing the heterogeneity of the operationalization of the concept
• Six community protective factors were identified: (1) school, (2) informal support from an extrafamilial adult, (3) involvement in extracurricular activities, (4) neighborhood, (5) community services, and (6) child protection services.
• At the school level, school organization and climate and school connectedness showed the most consistent results, with three studies out of four showing positive results.
• At the neighborhood level, neighborhood quality was the most consistent protective factor since three studies out of four reported positive outcomes.
• Overall, we found conflicting outcomes regarding the association between community protective factors and resilience in maltreated children. Further studies are necessary.

**Table:** *Implications for practice, policy and research*.

**Implications for practice**
• Some community factors appear to mitigate the negative consequences of maltreatment and even promote well-being.
• Identifying these factors and understanding how they come into play can inform practitioners on how to best help youth exposed to maltreatment.
• Leveraging protective factors at the community level in populations would allow for a broader reach than child-focused interventions, as children and families who are privileged enough to receive specialized and professional help in the face of adversity would not be the only ones to benefit from it.
• It can be postulated that changes within community institutions could foster a more sustainable impact on abused children.
**Implication for research**
• Experts in the field should operationalize and standardize the concept of resilience and community factors to allow for a fair assessment of the impact of factors on the resilience of maltreated children.
• Studies should rely on composite scores of resilience or on person-centered approach to observe distinct patterns of adaptation from multiple indicators.

## Results

A total of 9,553 articles were retrieved from two electronic databases based on the selected keywords. After reading the title and abstract, 238 studies were identified as potentially eligible. An additional 39 studies were identified through the snowball technique. A total of 194 articles were excluded as the inclusion criteria were not met. The remaining 44 studies were included in the final sample.

### Quality Assessment of the Included Studies

According to the quality assessment of the studies included in this review, 14 (31.8%) of the 44 studies were of good quality, as evidenced by a low level of risk of bias. Forty-one studies (93.2%) included total sample sizes of at least 100 participants, whereas the three remaining had sample sizes ranging from 37 to 99 participants. Nineteen studies relied on a longitudinal design. Thirty studies distinguished between the different forms of maltreatment, either by studying a sample comprised of participants who experienced a specific type of maltreatment or by reporting results separately for each form of maltreatment.

### Resilience/Positive Adaptation Rates

Of the 44 studies included in this review, 18 studies reported the prevalence of resilient participants in their sample. Considering that the studies used divergent methodologies to identify a resilient profile, the variability of this prevalence is very large (1.5%−66.7%). An outlier was identified and excluded from the range, since the prevalence in this study was between 72% and 92% (Hildebrand et al., 2019).

Of the 44 eligible studies, 27 conceptualized resilience as displaying fewer psychological symptoms than other participants considered as non-resilient, 5 conceptualized it as showing more positive functioning than other respondents considered as non-resilient, and 12 studies considered resilience as a combination of fewer levels of symptoms and a more positive adjustment than their counterparts. It is important to note that several studies are part of the same larger project and therefore the samples are not necessarily independent. The samples of nine studies are extracted from the *National Survey of Child and Adolescent Well-Being I and II* (NSCAW) and four are drawn from the *Longitudinal Studies on Child Abuse and Neglect* (LONGSCAN). Also, three studies use the sample of a summer camp project and two studies utilize the sample from the *Search Institute’s Profiles of Student Life: Attitudes and Behaviors survey*. The project affiliation of each of these studies is reported in Table 1.

### Factors Associated With Resilient Outcomes

To synthesize the community factors associated with positive adaptation, we grouped factors into different domains, institutions, or community spheres. The identified domains were: 1) school, 2) informal support from an extrafamilial adult, 3) involvement in extracurricular activities, 4) neighborhood, 5) community services, and 6) child protection services. Each domain is presented in succession and is further divided into subgroups. This method of data synthesis was selected to offer a concise and circumscribed overview of the findings for particular variables and to compare the findings among studies.

#### School factors

Of the 44 studies included in this review, 18 examined various school-related protective factors. Although the terminology of the variables varied in the studies, we classified them into four categories: school organization and climate (4 studies), school connectedness (4 studies), school stability (1 study), and educational and social support (10 studies). It should be noted that most of these results are based on a subjective perception of the school environment reported by participants.

##### School organization and climate

Some authors have explored school organization and climate, referring to the extent to which school is perceived as a safe and caring environment. The results of the longitudinal study conducted by Chapple and Vaske (2010) revealed that perceiving a better school organization (examples of items: “How well school maintains order,” “How well is the safety at school”) was associated with fewer school problems requiring parent visits among 1,080 children who experienced educational, emotional, and physical neglect. In a cross-sectional study among 3,281 maltreated adolescents, youth who perceived a better school climate were less likely to engage in excessive drinking, smoking, drug use, sexual activity, antisocial behavior, and suicide attempts, and were more likely to do well in school than youth who reported a less favorable school climate (Perkins & Jones, 2004). In a separate cross-sectional study by the same first author, with a sample of 18,592 physically abused adolescent girls, results revealed that when the school climate was perceived as better, the likelihood of eating disorder behavior (purging) decreased (Perkins et al., 2002). In both studies, school climate was assessed by items about whether the child “gets a lot of encouragement at school” or “likes school” and both studies were based on the same sample. Perceived school safety, measured with items such as “I feel safe going to and from school,” was found to be significantly associated with lower levels of suicidal ideation and attempts in a cross-sectional study with a sample of 8,592 adolescent girls and boys who were sexually abused (Eisenberg et al., 2007). All four studies have relied on homemade scales to assess perception of school organization and climate.

##### School connectedness

Other studies explored variables related to school connectedness, which is measured by a feeling of connection and belonging toward teachers and school (Dang, 2014). Dang (2014) found that school connectedness (an example of an item is “You feel part of your school”) predicted a lower level of psychological distress in a cross-sectional study with a sample of 150 homeless maltreated youth. Similar results were obtained in two other cross-sectional studies. Pérez-González et al. (2017) reported that school connectedness predicted lower levels of externalizing and internalizing behavior problems among 1,105 sexually victimized adolescents and Asgeirsdottir et al. (2010) discovered that positive attitudes toward school, which was conceptualized similarly as school connectedness, predicted a lower level of depressed mood and anger in a sample of 9,113 sexually abused adolescents. In the study, having positive attitudes toward school was a more influential predictor than parental support. However, a cross-sectional study conducted with 226 eighth graders, 81 of whom were victims of maltreatment, showed inconsistent results (Sagy & Dotan, 2001). The authors found that school membership, measured with items such as “I take part in many school activities” and “People here know that I can do good work,” predicted the level of perceived competence in the non-maltreated group of adolescents, but not in the maltreated group. School membership was not a predictor of adolescent psychological distress in either group (Sagy & Dotan, 2001). The four studies used different measures of school connectedness, two of which appeared invalidated or unpublished.

##### School stability

One cross-sectional study explored school stability, which refers to whether the child changed schools over their school years, among a sample of 99 sexually abused adolescent girls in the foster care system (Edmond et al., 2006). The authors found no significant differences in terms of school stability between the symptomatic and resilient adolescent girls. Resilience was defined in the study as the absence of psychopathology.

##### Educational and social support

Several studies have explored the sources of support received in a school context. Go et al. (2017), in a cross-sectional design with a sample of 130 maltreated children, observed that youth who reported having educational support in their school were less likely to display conduct problems. The association between educational support and anger control problems, however, was not significant. Results from Lemkin et al.’s (2018) longitudinal study among 318 maltreated children revealed that youth who reported having a supportive adult in school were more likely to graduate and participate in a school club. In terms of teacher support, only one cross-sectional study with a sample of sexually abused adolescents found significant results. Eisenberg et al. (2007) highlighted that teacher caring was significantly associated with lower levels of suicidal ideation and attempts in both sexually abused adolescent girls and boys.

Several studies showed inconclusive results. Teacher support was not predictive of risk behaviors in a longitudinal study of 110 maltreated children (Taussig, 2002) and child or caregiver perceived depression, anxiety, anger, and aggression in a cross-sectional study among 37 physically abused children (Ezzell et al., 2000). However, when teacher support was examined as a categorical variable (low, moderate, and high support), low teacher support predicted less resilience in a sample of 100 maltreated children (Hildebrand et al., 2019). This cross-sectional study referred to resilience as the individual capacities such as the sense of control, the relationship skills, and the emotional reactivity. The cross-sectional study of Aydin et al. (2016) revealed that teacher social support did not predict depressive symptoms and child post-traumatic stress reaction in a sample of 182 children and adolescents. However, participants with no or mild levels of depression reported more support from their teachers than participants with clinical levels of depression (Aydin et al., 2016). Other studies also underscored conflicting results for a similar concept. In a cross-sectional study conducted by Kassis et al. (2018), having a close relationship with a teacher was not a significant predictor of the level of resilience in a sample of 1,184 maltreated children, whereas in Choe’s (2021) longitudinal study, the student–teacher relationship was significant only at the cross-sectional level. The student–teacher relationship did not predict the level of dropout longitudinally in this sample of 1,316 maltreated seventh to ninth graders.

Finally, Chandy et al. (1996) examined the impact of the presence of a school clinic or nurse on the resilience of 1,011 sexually abused adolescent girls. In this cross-sectional study, resilience was defined as good school performance and absence of pathology and behavior problems such as suicidal ideation, eating disorders, sexual behaviors, pregnancy risks, and substance abuse. The authors found that resilient girls were more likely to report the presence of a nurse or clinic in their school.

#### Informal support from an extrafamilial adult

Extrafamilial adult support is also a widely explored community variable, as it was listed in seven studies. This type of support may include support from a coach, neighbor, family friend, camp counsellor, or other professional. In some studies, the identity of the adult in relation to the child was not specified and/or included multiple adults from outside of the family. Hence, in some cases, teacher support may have been included along with other adults from outside the family in this variable. We categorized the study as extrafamilial adult support since it cannot be inferred that the child responded to the questionnaire with a teacher in mind exclusively.

Two longitudinal studies taking place at a summer camp specifically explored the relationship between camp counselors and children. One study showed that a good relationship with a counselor significantly predicted adaptive functioning in a sample of 213 children among which 133 were maltreated (Cicchetti & Rogosch, 1997). When other individual and familial protective factors were included in the model, the quality of the relationship with the counselor was no longer significant for maltreated children but remained significant for non-maltreated children (Cicchetti & Rogosch, 1997). The second longitudinal study, focusing specifically on Latino children victims of maltreatment (*n* = 76), found that the relationship with a counselor was a predictor of resilient functioning (Flores et al., 2005). A better relationship with the counselor was related to greater levels of resilience in Latino children victims of maltreatment. When divided in subtypes of relationships, a warm relationship with the counselor was associated with greater levels of resilience, while a conflicted or closed/troubled relationship predicted a decrease in resilience (Flores et al., 2005). These two studies were part of the same research project and used the same validated instrument completed by camp counselors.

Five studies investigated support received from adults whose roles in the community were not specified and the results showed inconsistencies. A cross-sectional study previously introduced in this review (Eisenberg et al., 2007), found that other adult caring was significantly associated with lower levels of suicidal ideation and attempts in a sample of 8,592 maltreated adolescent girls and boys. In a cross-sectional study conducted by Feiring et al. (1998) with 154 maltreated children aged 8 to 15, adult support was not significantly related to depressive symptoms, self-esteem, and trauma symptoms. Pharris et al. (1997), in a cross-sectional design, found that adult caring and tribal leader caring acted as protective factors against suicidal ideation in a sample of 1,157 sexually abused American Indian Alaskan Native maltreated youth. These variables were also identified as protective factors against hopelessness in sexually abused girls but not in sexually abused boys. However, adult caring and tribal leader caring were not predictive of suicidal attempts in sexually abused adolescents in general (Pharris et al., 1997). In the cross-sectional study of Perkins and Jones (2004) previously described in this review, the results suggested that maltreated adolescents who displayed support from another adult were more likely to report helping others than those who mentioned lower other adult support. However, they were also more likely to exhibit heavy drinking, tobacco use, drug use, sexual activity, and suicide attempts and less likely to report success in school than those who experienced lower other adult support (Perkins & Jones, 2004). Another study from the same author also showed similar results. The likelihood of purging in physically abused girls increased when they reported more support from an adult (Perkins et al., 2002).

#### Extracurricular activities

Participation or attendance in extracurricular activities was examined in 10 articles. Three focused specifically on attendance in religious services, and seven on involvement in general activities, such as sports and clubs. The protective role of involvement in extracurricular activities was found in a cohort study with a sample of 1,493 emotionally and physically maltreated youth (Khambati et al., 2018). For children who experienced emotional maltreatment, engagement in extracurricular activities was significantly related to good educational attainment and greater well-being, but not to children’s self-esteem. For physically abused children, engagement in extracurricular activities was significantly related to educational attainment only (Khambati et al., 2018). In the study of Perkins and Jones (2004) previously mentioned, maltreated adolescents who stated participating in more extracurricular activities were less likely to have experienced tobacco use and purging and more likely to succeed in school and help others compared to adolescents who mentioned lower participation in extracurricular activities. However, the findings also revealed that maltreated adolescents who participated in more extracurricular activities were more likely to exhibit antisocial behaviors than those who displayed lower participation in extracurricular activities (Perkins & Jones, 2004).

Some studies have examined different types of activities, specifically. In a longitudinal study with a sample of 318 maltreated youth, participating in extracurricular activities in general had no impact on graduation rates (Lemkin et al., 2018). However, maltreated children who reported being involved in a school club specifically were more likely to graduate than children who mentioned participating in other kinds of clubs (sport team; drama, music, and dance or another performing arts group; scout troop; volunteer group; and religious group [Lemkin et al., 2018]). Asgeirsdottir et al. (2010), already described above, focused on sports participation among sexually abused adolescents. Sports participation was a stronger predictor among boys than for girls as it predicted a lower level of depressed mood, but was not predictive of anger (Asgeirsdottir et al., 2010). It is important to note that self-esteem partially mediated the relationship between sport participation and depressed mood (Asgeirsdottir et al., 2010). Kwak et al. (2018) conducted a longitudinal study among a sample of 790 maltreated adolescents and assessed the impact of being involved in specific activities on psychological symptoms and school engagement. Although the effect size was small, adolescents who participated in mentored groups, sports clubs, and academic clubs showed higher levels of school engagement compared to those who did not participate in these kinds of clubs (Kwak et al., 2018). Similarly, participation in academic clubs was related to lower levels of depression. However, engaging in mentored groups and sports clubs had no significant effect on depression symptoms, delinquency, and trauma symptoms. Involvement in academic clubs had no significant effect on delinquency or trauma symptoms and participating in art and music clubs had no significant effect on school engagement, depression symptoms, and delinquency. Contrary to expectations, the authors also found that some extracurricular activities were associated with poorer adjustment. Indeed, adolescents who participated in art and music clubs tended to demonstrate higher levels of trauma symptoms compared to those who did not participate in these kinds of clubs (Kwak et al., 2018).

In the 18-month longitudinal study carried out by Leon et al. (2008), club membership had no direct effects on sexually ruminative thoughts in a sample of 142 maltreated children. However, the interaction between club participation and sexual abuse severity was significant. For children who experienced less severe sexual abuse (i.e., fewer acts of sexual abuse), club membership was associated with fewer sexually ruminative thoughts.

A few studies focused on the association between attendance to religious or traditional services and positive adaptation in maltreated youth. Contrary to their expectations, Edmond et al. (2006) observed no differences between non-resilient maltreated girls and resilient maltreated girls in terms of attendance to religious services. Kim (2008) found similar results in their cross-sectional study. Frequency of attendance to religious services was not associated with internalizing and externalizing behaviors among a sample of 384 maltreated and non-maltreated children. However, an interaction was found between the level of attendance in religious services and externalizing behaviors in boys. A high attendance to religious services was predictive of fewer externalizing behaviors in non-maltreated boys (*n* = 196), but not in maltreated boys (*n* = 188). Pittenger et al.’s (2018) longitudinal study even indicates that it could be considered as a potential risk factor in a sample of 1,664 maltreated youth, as the results revealed that those with a greater involvement in religious services at Wave 1 (11–17 years old) were more likely to use marijuana approximately 3 years later. Pharris et al. (1997) examined the impact of involvement in traditional activities on hopelessness and suicidal behaviors of American Indian Alaskan Native maltreated youth, as mentioned previously in this review. Their findings revealed that involvement in traditional activities protected against suicidal attempts in maltreated boys, but not among maltreated girls (Pharris et al., 1997).

#### Neighborhood and community factors

In total, 16 studies explored protective factors related to neighborhood characteristics. Many slightly different variables in terms of terminology and conceptualization were considered. In the pursuit of parsimony, they were divided into two main categories: neighborhood organization and climate (including quality and security; 12 studies), and collective efficacy (4 studies).

##### Neighborhood organization and climate

Neighborhood characteristics include different constructs, such as quality, safety, and organization, which are widely studied as community protective factors. The majority of studies has examined neighborhood safety. Perceived neighborhood safety was associated with fewer behavior problems in a cross-sectional study of 168 maltreated children (Maschi et al., 2010). However, the association between maltreatment history and behavior problems remained significant even though neighborhood safety protected against maladaptive functioning of the children. Yoon et al. (2020) conducted a longitudinal study over 18 months with 711 maltreated adolescents. Three groups emerged from the multinomial logistic regression model: adolescents who moved from the lesser resilience to the greater resilience group, participants who remained in the greater resilience group and adolescents who moved from the greater resilience group to the lesser resilience group. The authors found positive results in that youth who perceived their neighborhood as safer were more likely to remain in the greater resilience group (Yoon et al., 2020). In the same vein, a cross-sectional study involving 589 maltreated youth aged 10 to 17 discovered three psychological adjustment profiles through cluster analysis: the Well-adjusted group, the Moderately adjusted group, and the Struggling group. An adequate psychological adjustment was characterized by less depressive symptoms and a higher level of self-efficacy and problem-solving attitudes. The results highlighted that the Well-adjusted group lived in safer neighborhoods than those in the two other groups (Sianko et al., 2016).

In other studies, neighborhood security did not yield any significant results. In a cross-sectional research, neighborhood safety did not predict the level of behavior problems in a sample of 237 child victims of sexual abuse (Williams & Nelson-Gardell, 2012) and it was not associated with the trajectory of academic functioning in a sample of 1,776 maltreated children (Holmes et al., 2018). Yoon et al. (2018) also conducted a longitudinal study among 350 maltreated children. Contrary to their expectations, neighborhood safety and levels of problems within the neighborhood were not associated with the levels of externalizing behaviors, internalizing behaviors or post-traumatic symptoms in this sample. In a separate study by the same first author, neighborhood safety also was not associated with the level of child aggressive behaviors in a sample of 499 maltreated children drawn from the first wave of the greater American survey NSCAW-I. However, fewer problems with the neighborhood were associated with lower levels of aggressive behaviors in maltreated children (Yoon et al., 2017).

A few studies have focused on neighborhood quality as a potential predictor of positive adaptation despite the experience of child maltreatment. Neighborhood quality was found predictive of the level of resilience in a longitudinal study with a sample of 1,193 maltreated children (Sattler & Font, 2018). Similar findings were discovered in a cross-sectional study with a sample of 597 12-year-old children (Martinez-Torteya et al., 2017). The authors relied on a person-centered approach and identified five latent profiles: Consistent maladaptation, School maladaptation/family protection, Low socialization skills, Posttraumatic stress problems, and Consistent resilience. The authors defined resilient children as exhibiting less behavioral problems, emotional problems, and posttraumatic stress symptoms and demonstrating higher levels of competence in socialization, communication, daily living skills, motor skills, daily activities, social activities, and school performance and maintaining a good relationship with their parents. Findings revealed that caregivers of maltreated children in the resilience group perceived the quality of their neighborhood as better than caregivers of children in the other four groups (Martinez-Torteya et al., 2017). Positive community environment was also explored through a person-centered approach. Oshri et al. (2017) conducted a longitudinal study among 1,179 maltreated adolescents. Through a growth mixture model, the authors identified four classes of adaptation, including two exhibiting resilience (Stress-resistant, Emergent Resilience) and two demonstrating little resilience (Breakdown, Unresponsive-maladaptive). Authors described resilience as showing less risk behaviors (delinquency, substance use, and trauma and depressive symptoms) and a positive adjustment (future expectations, better grades, life skills, and better physical health). In the Emergent Resilience class, no relationship was found between the community environment and their profile of adaptation. However, participants in the Stress-resistant class were more likely to report a positive community environment.

Aside from neighborhood safety and quality, other variables were considered in the articles identified in the review. Community connectedness, examined by Pérez-González et al. (2017), was not significantly related to reduced externalizing and internalizing behaviors among sexually abused adolescents. Chapple and Vaske (2010), already introduced in a previous section, used a more global construct of neighborhood organization that included social cohesion, access to institutional resources, and informal as well as formal social control. Neighborhood organization was not significantly related to behavioral school problems. Contrary to what was expected, among participants who experienced moderate levels of emotional neglect, the odds of school suspension were higher for those who lived in well-organized neighborhoods than those who lived in poorly organized neighborhoods. Neighborhood organization did not influence the probability of school suspension for children who experienced low and high levels of emotional neglect, nor did it moderate the effect of child neglect on educational problems in emotionally neglected children.

##### Collective efficacy

Collective efficacy as a factor related to resilience was examined in four studies. This concept is generally defined as social cohesion (or solidarity) combined with neighbors’ willingness to intervene for the common good (otherwise coined as informal social control; Sampson et al., 1997). In a 2-year longitudinal study of a sample of 1,354 bereaved maltreated youth, Sasser et al. (2019) found that youth who perceived higher collective efficacy were more likely to experience fewer externalized behavior problems. The cross-sectional study conducted by Yonas et al. (2010) with a sample of 786 maltreated children with an average age of 12 years revealed that collective efficacy did not moderate the relationship between physical or sexual abuse and externalizing behavior problems, but significantly moderated the relationship between neglect and externalizing behavior problems. A higher score on the collective efficacy scale was associated with lower externalizing behavior scores in neglected youth (Yonas et al., 2010). The association was not significant for non-neglected youth, which suggests that collective efficacy may be a protective factor of externalizing behaviors in neglected youth specifically.

Two studies examined the two components of collective efficacy separately. Jaffee et al. (2007) conducted a longitudinal study with 2,181 physically abused children and found that those who lived in neighborhoods with higher informal social control were more likely to be resilient (i.e., less likely to have emotional and behavioral problems and more likely to have better reading skills) than children living in neighborhoods with lower informal social control. However, in a cross-sectional research by Emery et al. (2015), while neighborhood social control was not related to internalizing behavior problems in a sample of 509 physically abused children, it was unexpectedly associated with greater externalizing behaviors. Living in a neighborhood with higher social cohesion was associated with higher levels of resilience in maltreated children in the study of Jaffee et al. (2007), but no association was found with internalizing and externalizing symptoms in the study of Emery et al. (2015).

Emery et al. (2015) also investigated informal social control specifically related to child maltreatment (i.e., willingness to intervene when witnessing child maltreatment). It was examined in terms of protective (e.g., de-escalate the situation to protect the child) and punitive (i.e., invoking social sanctions to deter further abuse) informal social control. While punitive informal social control was not predictive of externalizing and internalizing behavior problems, protective informal social control was related to less externalizing behavior problems. Moreover, child victims of very severe physical abuse who reported higher protective informal social control were less likely to exhibit externalizing behaviors. However, the relationship between protective informal social control and internalizing behaviors was not significant.

#### Community services

Among the 44 reviewed studies, three studies investigated the use of services offered by the community or institutions as a potential correlate of resilience. In the study conducted by Oshri et al. (2017) described previously, results showed that participants in the Emergent Resilience class and the Stress-resistant class were more likely to report having received services following a caseworker visit than the Unresponsive-maladaptive trajectory children. Youth who displayed greater resources, including those who received services, were more likely to belong to the Emergent Resilience class than the Unresponsive-maladaptive class and exhibited lower levels of risk outcomes such as delinquency behaviors, substance use, and depressive and trauma symptoms (Oshri et al., 2017). Conflicting results were found in the Yoon et al. (2020) study, as maltreated youth who received behavioral services were less likely to remain in the greater resilience group over 18 months. In the cross-sectional study by Sianko et al. (2016) discussed previously, community services were not predictive of adjustment in a sample of maltreated adolescents. Studies examining these protective factors all relied on homemade items.

#### Child protection services

Five studies examined child protection services. Two focused on the type of placement, while one examined both the impact of placement stability and the support of a caseworker agency (Lutman & Farmer, 2013). Otherwise, one study specifically examined placement stability and another one, the caseworker agency support. Two longitudinal studies examined the impact of placement type on positive adjustment and reported nonsignificant results in a study with maltreated adolescents in general (Yoon et al., 2020) and in a sample of 86 sexually abused adolescent girls (Daigneault et al., 2007). Two studies explored the effects of placement stability. Lutman and Farmer (2013), in a 5-year longitudinal study with 138 neglected children, found that those who lived stably away from home were more likely to show greater well-being than those who lived in unstable environments. Proctor et al. (2010) used growth mixture modeling with a sample of 279 maltreated children followed up over 8 years to identify classes of adaptation for internalized and externalized problems. For both externalized and internalized problems, caregiver stability predicted membership to the stable positive adjustment classes (i.e., youth scoring in the normal range of externalizing or internalizing behaviors over 8 years).

Two studies focused on the support of a practitioner or a caseworker. In Lutman and Farmer’s (2013) longitudinal study, children who were in an out-of-home placement were more likely to show greater well-being, when their parents had received a specialist or informal support in view of the reunification. In the second study, the caseworker agency support had an indirect effect on the sexually ruminative thoughts among the sample of 142 abused children (Leon et al., 2008). For youth with greater caseworker agency support, the level of ruminative thoughts remained stable even when the level of sexual abuse severity increased (i.e., when the participant reported experiencing multiple sexual abuse behaviors). However, in youth who reported less caseworker agency support, higher severity of abuse was predictive of more sexually ruminative thoughts.

## Discussion

Until recently, the study of individual and familial factors as predictors of positive adaptation in maltreated children has been at the forefront of the resilience literature, at the expense of the exploration of community factors. This review aimed to (1) quantify the resilience rates of maltreated youth in the selected studies, (2) review the community protective factors of resilience of maltreated youth in the literature, and (3) assess the quality of the studies using a standardized coding process. To our knowledge, this is the first systematic review to focus solely on community factors that may promote resilience in maltreated youth.

Of the 9,553 studies identified, 44 met the prespecified criteria and were included in this review. Most studies measured resilience as the absence of clinical levels of difficulties or symptoms of psychopathology. However, the clinical cut-off from which one is considered resilient is sometimes arbitrary and varies from one study to the other. Among these studies, some labeled children as resilient when they showed positive adaptation on a single indicator (e.g., Williams & Nelson-Gardell, 2012), whereas others conceptualized resilience across multiple contexts (e.g., Oshri et al., 2017; Perkins & Jones, 2004). Only 5 studies out of 44 defined and measured resilience as the presence of positive adaptation, such as self-esteem, well-being, or educational attainment (e.g., Khambati et al., 2018). This highlights the fact that positive adaptation is mainly viewed as the absence of negative indicators (i.e., absence of psychopathology), rather than in positive terms (i.e., happiness, health, and well-being). A broader understanding of resilience that is based on both positive and negative indicators needs to be reinforced, especially since well-being and suffering are not necessarily mutually exclusive (Masten, 2018). Such heterogeneity in the operationalization of resilience makes it difficult to assess and compare the prevalence of resilience across studies and populations, as evidenced by the wide range of 1.5% to 66.7% obtained.

Identification of community factors promoting positive adjustment of children and adolescents exposed to maltreatment is valuable for designing intervention and prevention initiatives, as well as in guiding public health policies. In this review, we identified six categories of community protective factors: (1) school, (2) informal support from an extrafamilial adult, (3) involvement in extracurricular activities, (4) neighborhood, (5) community services, and (6) child protection services. Within these categories, the most studied variables were the neighborhood organization and climate, as well as the educational and social support. While about 20 studies have evaluated these variables, the results remain inconclusive as about half of the studies show nonsignificant results (e.g., Aydin et al., 2016; Yoon et al., 2018).

At the school level, the variables leading to the most consistent results were school organization and climate and school connectedness, with three out of four studies showing positive outcomes, such as school success, and fewer internalizing (e.g., suicidal ideations) and externalizing (e.g., substance use, purging) difficulties. However, these results should be interpreted with caution since only four studies have explored these concepts and relied on nonvalidated, homemade measures. Evidence was also less robust regarding the other school variable, as school stability was the least studied variable with a sole study addressing it.

The informal support from an extrafamilial adult and the extracurricular activities factors show mostly promising results. However, two studies on adult support and one study on club participation gave rise to unexpected results as a higher level of these variables was associated, in turn, to higher levels of risk behaviors such as tobacco use, heavy drinking, or purging (Kwak et al., 2018; Perkins & Jones, 2004; Perkins et al., 2002)

In the neighborhood organization and climate category, neighborhood quality was more consistently associated with positive adaptation (i.e., in three out of four studies). Even though neighborhood safety has been evaluated in seven studies, half of them led to nonsignificant findings. Moreover, neighborhood connectedness failed to be associated with resilience, and neighborhood organization was instead predictive of increased symptoms. The four studies that examined collective efficacy showed contrasting results. However, one study suggests that collective efficacy may have a more specific impact on neglected children (Yonas et al., 2010). Although limited, the evidence also suggests that community services, type of placement, placement stability, and caseworker agency support are promising protective factors. However, more studies are needed to better assess their impact on the resilience of maltreated children.

Notwithstanding the categories, we found conflicting results regarding the relationship between community factors and the resilience of maltreated youth in general. While most results showed a significant relationship between protective factors and positive adaptation or a decrease in symptoms, others failed to find an association or even revealed an opposite relationship, indicating that the variable rather acted as a risk factor. The fact that the impact of community variables is still unclear, and that studies focus on general maltreatment, does not allow for the identification of factors relevant to the resilience of children experiencing a particular trauma.

### Recommendations for Future Studies

This systematic review highlights the bias and the methodological shortcomings of some studies (concept measured by an item, weak internal consistency of the scales, management of missing data, sample size, and representativeness of some nested samples) and the choice of statistical analyses (unmeasured cofounding variables and low statistical power). Furthermore, our review underscores the overrepresentation of Western communities in the studies conducted and highlights the need to better represent the Eastern realities. With this state of knowledge in mind, several recommendations for the design of future studies can be mentioned.

Above all, to improve the field of resilience, longitudinal designs should be favored since resilience, when defined as a dynamic concept, should not be measured only at one point in time if one wants to assess it as a process. Moreover, a cross-sectional design does not allow for a definitive determination of the temporal sequence between the variables studied. The wide discrepancies in rates across studies illustrate the lack of consensus regarding the operationalization of resilience and underscores the need to unify definitions of this concept. Resilience is a complex phenomenon that requires a standardized and consensual definition by a panel of experts in the field, as well as guidelines to adequately study it.

Future studies exploring community variables should also attempt to better delineate the multiple constructs. Confusion about the terminology of some similar constructs was evident, and particularly frequent among neighborhood variables. For instance, neighborhood quality was assessed by multiple studies, all of which had their own definition. In some cases, it reflected a combination of distinct factors, such as quality of life and perceived neighborhood safety whereas other authors failed to provide a conceptual definition. Moreover, some variables’ names did not align with the scale used to measure the construct. In addition, future studies should benefit from using standardized and validated measures as very few studies relied on the same scale. The ones that did stem from the same larger research project and thus had their data drawn from the same sample, which greatly limits the generalizability of the findings.

Moreover, we found that community protective factors were assessed by a variety of informants (e.g., children, parents, teachers, caseworkers, etc.). Most community factors were assessed by a single informant, while a limited number were measured objectively (i.e., placement and school stability, participation in extracurricular activities, and the presence of a nurse at school). Child reports were used, in a majority of cases, to assess school-related variables, support from an extrafamilial adult, and participation in extracurricular activities, whereas caregiver reports were more frequently employed for neighborhood variables. It should be noted that the informant reports are a matter of their own perceptions that varies accordingly to context; thus, different informant rating constructs commonly provide diverging estimates. A growing number of researchers call for relying on cross-informant measures and for considering the gap between the subjective impressions of two or more of these informants regarding the studied phenomenon. This allows for a more valid and comprehensive assessment of a construct.

### Limitations

This systematic review must be considered with its own limitations. First, it only includes articles in English or French and does not distinguish between the impact of resilience in children or adolescents, nor between girls and boys. Retrospective studies with adult participants were also excluded to avoid the introduction of biases related to the memory of the events. These studies might have been useful in retrospectively documenting the dynamic process of resilience over time. Finally, due to the complexity of this body of work, it was not possible to compare the impact of community factors according to the varying conceptualizations of resilience. Such an effort, however, would be relevant to inform the elaboration of a standardized definition of resilience.

### Practical Implications

This review reinforces the fact that poor adaptation following events as traumatic as maltreatment in childhood is not inevitable. Some factors appear to buffer negative sequelae and even foster well-being, and positive adaptation more generally. Identifying these factors and understanding how they come into play can greatly inform practitioners on how to best assist youth exposed to maltreatment.

From a research point of view, this review emphasizes the need to reach a common ground on the operationalization of resilience. Regarding community factors especially, it appears that examining resilience as positive adaptation on a single indicator or within a single context has limited clinical significance and that a resilience-across-multiple-contexts definition would be preferable. Unlike personal and familial protective factors which can be more easily targeted in a tailored intervention, modifying community factors would act more in a one-size-fits-all fashion. For instance, improving neighborhood safety is likely to benefit the entire community, whereas an intervention carried out with a child will benefit the child and his/her family more specifically. Transitioning to a more comprehensive assessment of resilience that encompasses multiple domains (educational, social, emotional, etc.) would be more enlightening for community workers and institutions. Studies should use composite scores of resilience or rely on person-centered approach to observe distinct patterns of adaptation from multiple indicators.

From a clinical perspective, optimizing protective factors at the community level would have a broader reach than child-focused interventions, because more families could benefit from it, especially those who cannot afford specialized professional help. It can also be posited that change within community institutions could promote a more sustainable impact on maltreated children. Moreover, not only could community factors contribute to resilience in children, they could also offer primary or secondary prevention of child maltreatment. For instance, social cohesion and informal social control were found to have deterring effects on the maltreatment of children (Maguire-Jack et al., 2021).

In conclusion, improving our understanding of environmental factors, including the community level, shifts the responsibility of resilience from an individual one to a collective one. As argued by Ungar (2011), resilience results from an interaction between individual and environmental factors, the latter being necessary to potentiate individual qualities that favors recovery from trauma. As this view of resilience is getting more accepted in the field, research efforts should be deployed accordingly.

## Supplemental Material

sj-docx-1-tva-10.1177_15248380221117234 – Supplemental material for A Systematic Review of Community-Level Protective Factors in Children Exposed to Maltreatment Click here for additional data file. Supplemental material, sj-docx-1-tva-10.1177_15248380221117234 for A Systematic Review of Community-Level Protective Factors in Children Exposed to Maltreatment by Arianne Jean-Thorn, Amélie Tremblay-Perreault, Valéry Dubé and Martine Hébert in Trauma, Violence, & Abuse

## Appendix A

### Search Strategy

Initially, in order to include as many studies as possible, we used the following search strings: child AND (abus* OR maltreat* OR neglect OR abandon* OR ill#treat* OR advers* OR trauma* OR ACE* OR victim* OR violen*) AND (resilien* OR protective OR adapt* OR adjustment) as keywords, title, or abstract. To refine our research, we then also included the following terms: societ* OR resource* OR environment* OR social OR education* OR service* OR context* OR macro* OR network OR support OR ecology OR collective OR neighbo* OR community OR peer OR demograph* OR extra* OR system* OR teacher*.

The identification of eligible articles through the first screening was done by the first author of this review. When the title or abstract did not provide enough information to validate whether the article was eligible or not, the study was included for the eligibility assessment.

### Quality Assessment

A data extraction sheet was created to collect the following information of each eligible study: general information (title, authors, year of publications, country of publication), sample (sample size, age, gender, research project), forms of maltreatment (sexual, physical, psychological or emotional abuse and neglect, investigated separately or all types combined), type of methodology (analyses, type of protective factor, and outcome measures), outcomes, limits, and study quality. Quality was assessed based on the *Standard Quality Assessment Criteria for Evaluating Primary Research Papers* (Kmet et al., 2004). This guide suggested 14 criteria, but we excluded three since the quasi-experimental nature of the research design with maltreated populations offers limited possibilities in terms of random allocation and blinding processes. The quality of the studies was measured by seven criteria assessing the clarity or the appropriateness of the objective, design, sample size, recruitment method, description of the sample and measures used, and choice of analyses. Four additional criteria assessed the presence of variance estimates and control variables, the completeness of the results, and the quality of the reported findings. These criteria could be coded as absent, partially present, or present.
