# Supplementary material for: A Systematic Review of Community-Level Protective Factors in Children Exposed to Maltreatment
Source: Trauma Violence Abuse. 2022 Sep 1;24(4):2827–42. doi: 10.1177/15248380221117234 (PMC10486159; doi:10.1177/15248380221117234)
Supplement: sj-docx-1-tva-10.1177_15248380221117234 – Supplemental material for A Systematic Review of Community-Level Protective Factors in Children Exposed to Maltreatment [file sj-docx-1-tva-10.1177_15248380221117234.docx]

Appendix A

**Search strategy**

Initially, in order to include as many studies as possible, we used the following search strings: child AND (abus* OR maltreat* OR neglect OR abandon* OR ill#treat* OR advers* OR trauma* OR ACE* OR victim* OR violen*) AND (resilien* OR protective OR adapt* OR adjustment) as keywords, title or abstract. To refine our research, we then also included the following terms: societ* OR resource* OR environment* OR social OR education* OR service* OR context* OR macro* OR network OR support OR ecology OR collective OR neighbo* OR community OR peer OR demograph* OR extra* OR system* OR teacher*.

The identification of eligible articles through the first screening was done by the first author of this review (MASKED FOR REVIEW). When the title or abstract did not provide enough information to validate whether the article was eligible or not, the study was included for the eligibility assessment.

**Quality assessment**

A data extraction sheet was created to collect the following information of each eligible study: general information (title, authors, year of publications, country of publication), sample (sample size, age, gender, research project), forms of maltreatment (sexual, physical, psychological or emotional abuse and neglect, investigated separately or all types combined), type of methodology (analyses, type of protective factor and outcome measures), outcomes, limits and study quality. Quality was assessed based on the *Standard Quality Assessment Criteria for Evaluating Primary Research Papers* (Kmet et al., 2004). This guide suggested 14 criteria, but we excluded three since the quasi-experimental nature of the research design with maltreated populations offers limited possibilities in terms of random allocation and blinding processes. The quality of the studies was measured by seven criteria assessing the clarity or the appropriateness of the objective, design, sample size, recruitment method, description of the sample and measures used, and choice of analyses. Four additional criteria assessed the presence of variance estimates and control variables, the completeness of the results, and the quality of the reported findings. These criteria could be coded as absent, partially present or present.
